# What it means to be an adult child of a person with dementia

**DOI:** 10.3402/qhw.v8i0.21676

**Published:** 2013-10-22

**Authors:** Annika Kjällman-Alm, Karl-Gustaf Norbergh, Ove Hellzen

**Affiliations:** Department of Health Sciences, Mid Sweden University, Sundsvall, Sweden

**Keywords:** Adult children, dementia, life world, phenomenological hermeneutics, qualitative research

## Abstract

The prevalence of dementia as a disease has increased worldwide with advancing age and growing population numbers, affecting whole families. However, most previous research does not separate the spouses or cohabitants from the adult children, but instead regards all next of kin involved in the everyday care of the person suffering from dementia as caregivers. This has made it difficult to find previous research regarding what it means to be an adult child of a person with dementia, and as such, the aim of this study is to explore that topic. The method used was narrative interviews analysed using phenomenological hermeneutics. Our comprehensive understanding showed that to be an adult child of a person with dementia means being burdened with the responsibility to act on behalf of the diseased parent despite a deep sense of grief and loss, which leads to frustration and despondence. The adult children's existence and reality are threatened not only by the loss of the parent but also by the possibility that one day they too may inherit the disease. This could be compared to a psychic crisis, which is defined as a situation that leads to radical changes in the afflicted person's relationship to life and reality, or, simply, “an upset in a steady state”. The findings suggest that adult children of people with dementia are in need of support for a substantial period of time in order to adapt to the fact that they have lost a parent who is still alive. They also need information about the disease and the process of diagnosis and treatment to feel more a part of the process, as well as understand the behavior and needs of their parent.

The prevalence of dementia diseases increases worldwide with advancing age and growing population numbers (Kalaria et al., 2008; Wimo, Winblad, Aguero-Torres, & von Strauss, 2003). The 2012 *World Alzheimer Report* estimated that 36 million people were living with dementia worldwide in 2010, and that this number will increase to 66 million by 2030 and 115 million by 2050. Low- and middle-income countries are the source of nearly two-thirds of these statistics, with the sharpest increases in numbers set to occur there as elderly populations grow (Batsch & Mittelman, 2012). Marc Wortmann, executive director of ADI, said, “Around the world a new case of dementia arises every four seconds. Our current health systems simply cannot cope with the explosion of the dementia crisis as we all live longer” (WHO, 2012).

In Sweden, about 25,000 persons are diagnosed with dementia each year, totaling 150,000 sufferers (SBU, 2006). After diagnosis, the need for support is high, but next of kin often feel abandoned and in need of emotional support (Neufield & Eastlick Kushner, 2009; Sanders, Ott, Kelber, & Noonan, 2008). Most previous research does not separate the spouses or cohabitants from the adult children but regards all next of kin involved in the everyday care of the person suffering from dementia as caregivers or states them as next of kin (Betts Adams, 2006; Laakkonen et al., 2008; Sanders et al., 2008; Vernooij-Dassen, Derksen, Scheltens, & Moniz-Cook, 2006). This makes it hard to find previous research regarding what it means to be an adult child of a person with dementia. However, it is clear that the role for next of kin changes to one of supervision and decision making. Feelings of frustration, sorrow, and distance were common as well as feelings of affection and benevolence toward the person suffering from dementia (Betts Adams, 2006; Laakkonen et al., 2008). In one study, adult children expressed apprehension for the sufferer regarding such problems of daily life as heating, falls, handling money, traffic safety, and not being able to find the way home when going for a walk. Another concern was cooking. Despite these concerns, many felt that the sufferer would be better off at home and did everything to make the environment safe for him or her, such as selling the car and installing different safety features on the heater, stove, and other devices (Gilmour, Gibson, & Campbell, 2003). However, the changes in the sufferers’ personality after the diagnosis are especially hard for next of kin and adult children to handle (Harman & Clare, 2006; Laakkonen et al., 2008; SBU, 2006; Strang, Koop, Dupuis-Blanchard, Nordstrom, & Thompson, 2006). In 2009, the Swedish Parliament passed a new law that states, “Municipalities are obliged to offer support to persons caring for people with chronic illnesses, elderly people, or people with functional disabilities” (Swedish Social Services Act, 2009). There is, however, no detailed description of the extent or kind of services to be provided, and the municipalities have extensive freedom in implementing the legislation (Jegermalm, 2003; Johansson, Long, & Parker, 2011).

Our proposition is that the personality changes offer a great challenge to the adult children. To see your father turning into a little boy or your mother into a schoolgirl should have an impact on the lifeworld of the adult children. To be able to look up to your parents as role models and use them as a template for how you deal with the world at any age is essential to the individual's personal growth (Erikson, 2004). In order to elucidate the situation for adult children of persons with dementia and help fill the knowledge gap that we feel exists, it is important to explore adult children's life situation from their perspective.

## Aim

The aim of this study was to explore what it means to be an adult child of a person with dementia. By deepening the understanding of this issue, it may be possible to access the specific need for support and information for the adult children.

## Methods

The study used a lifeworld approach in order to explore what it means to be adult children of persons with dementia. The lifeworld is our basic reality, and the world shows itself to our consciousness and is inexplicably linked to the perceiver of that reality (Bengtsson, 2001; Dahlberg, Dahlberg, & Nyström, 2008). To understand what shows itself to our consciousness (phenomenology), it has to be interpreted (hermeneutics); there is no understanding without explanation (Ricoeur, 1976). Therefore, to increase the understanding of being an adult child of a person with dementia, this study used phenomenological hermeneutics for the analysis (Lindseth & Norberg, 2004).

## Settings and participants

The participants consist of adult children from an ongoing evening support group conducted at a municipal support center. The support group is held at the municipal support center one evening a month by trained social workers who specialize in dementia care and next-of-kin support. All meetings are free of charge and are held at the center with trained group facilitators. A meeting usually lasts between 90 min and 2 h, including a coffee break. The participants are a part of the group for as long as they like and attend meetings when they can. Topics include brain function and what can influence function, dealing with memory loss, how to respond to a person suffering from dementia, and the latest research regarding dementia. Facilitators always start off the meetings by reminding everyone of the confidentiality of everything said in the group context.

The inclusion criteria for the study's participants were being part of an ongoing support group provided by the municipality and being willing to talk about their experiences before and during their parent's disease. The respondents were all approached at a support group meeting and verbally informed about the study and what it entailed. Written information and consent forms were also handed out to a total of 20 participants. Informed consent was received from nine participants, who were then interviewed at their convenience.

## Data collection

Data were collected from March to May 2012 and consisted of digitally recorded, open interviews with nine participants, eight females, and one male between the ages 35 and 65. The interviews were conducted at the support center by the first author since it was most convenient for the participants.

The interviewees were asked to narrate about their experiences before the parent was diagnosed with dementia, their experiences after diagnosis, and how it was now, with clarifying follow-up questions when needed such as “Can you give an example?” or “How did that make you feel?”. According to Ricoeur (in Wood, 1991), we should keep in mind that history is repeated but life is lived. His point is that the text is not finished by the storyteller but by the reader, and that recreates life through the story. More precisely, the meaning of the story is a mix of the text's world and the reader's world. The reading itself becomes the critical moment of the analysis and the story's ability to change the experience of the reader. The reading in a way finishes the text, transforming it to a guide for reading, with zones of indefinitiveness, and with a hidden richness of interpretation possibilities and the power to be reinterpreted in a new historical context (Ricoeur, *Life in Quest of Narrative* in Wood, 1991). It was therefore important to let the interviewees narrate openly and not interfere with questions but rather let them finish their train of thought. The interviews lasted between 60 and 90 min and were transcribed verbatim by the interviewer. In order to ensure confidentiality, all personal information was replaced by codes, and codes and transcripts were stored in a safe accessed only by the interviewer. Personal information and codes were kept separate.

## Ethical considerations

The study was conducted after approval from the Ethics Committee of the Medical Faculty, Umeå (Dnr 2011-376-31M). The respondents could at any time drop out of the study without giving a reason or affecting their place in the support group. However, interviewing can cause emotional upset for the individual; therefore, the interviews took place in an open and nonjudgmental atmosphere. Pauses for crying or having a glass of water were fine. We also received approval from the head of the municipal counsel on social care, which enabled us to inform participants and recruit participants in the ongoing support group as well as use the facilities for interviews. Personnel at the center were also on call for emotional backup after the interview should the interviewees need it. The personnel at the center have experience in dealing with individuals in emotional distress, and they are well known to the interviewees. However, no incident that required backup from personnel occurred during the study.

## Summary of the process of analysis

In order to interpret and illuminate what it means to be an adult child of a person with dementia as experienced by our respondents, a phenomenological hermeneutical research that was influenced by Paul Ricoeur (1976), and described by Lindseth and Norberg (2004), was used. The lived experience remains private, but its meaning can be made visible. To make the meaning visible requires an interaction between the text and the reader where the reader interprets the text. Interpretation is a special form of understanding; it is based on all forms of written expressions of life. The text remains mute, and an asymmetrical relationship evolves where only one speaks and the author's intention is unclear for the reader. By understanding and interpreting the text, the reader is allowed to access all possible worlds and a possibility to orientate oneself in those worlds (Ricoeur, 1976).

The analysis began with a naïve reading, progressed to explanatory structural analysis, and ended in a new understanding or comprehension. During naïve reading, the whole text was read in order to grasp a first understanding, verbalized as a naïve understanding of what it means to be an adult child of a person with dementia. The first interpretation during the naïve understanding can be seen as pure guesswork (Ricoeur, 1976), and the structured analysis viewed as a validation of the guesswork and a way to explain the text (Lindseth & Norberg, 2004).

The whole text was then structurally analyzed with the naïve understanding in mind. The whole text was divided into meaning units (see Table I). The meaning units were condensed and compared with respect to differences and similarities, and they were finally abstracted into subthemes and themes. The themes and subthemes were then reflected upon against the naïve reading to validate the findings. Finally, the naïve reading, structural analysis, relevant literature, and authors’ pre-understandings were brought together to develop a new comprehensive understanding of what it means to be an adult child of a person with dementia. The authors’ pre-understandings consist of working as registered nurses in dementia care as well as previous studies in dementia care settings. However, Lindseth and Norberg (2004) state that the pre-understanding is not bracketed but rather our judgment about the factual, about what is the case, in order to become open to our own experience and to the understandable meaning implicit in this experience (Lindseth & Norberg, 2004).

**Table I:** Examples from the structural analysis of meaning units and their corresponding condensation.

Meaning units	Condensation
He went to bed when eh … well early in the evening and was up during the night and the neighbors started calling. I had no idea because I was there during the day … and then she told me on one evening she called that dad went down to her and I had been home maybe a couple of hours, and at that time everything was ok.	He went to bed early and was up at nights. I had no idea until the neighbors called. Everything was fine during the day when I was there.
Then she didn't know where she was, if she walked the same way every time it was fine … then she found her way home…. So it was awful, it was really awful. You went completely blank. I'm lucky I have my husband … because every time the phone rang I thought*—*My God what has happened, is she run over or what?!	She did not know where she was if she did not walk the same way. It was awful, every time the phone rang you expected the worst.
No, but it's kind of odd this … losing your mother … and becoming mother for you own mother in a way, and mother to your father as well sort of. I can say that it brings matters to its head.	It is weird, losing your mother and becoming a parent to your parents.

## Findings

### Naïve reading

To be an adult child of a person with dementia means to be compelled to take the parental role vis-à-vis your mother or father with all that this entails: personal hygiene, economy, and safety. At the same time, you mourn the loss of that parent and long for them to return, and to feel close to them again. You are also faced with working double shifts to take care of your parents’ household as well as your own, making sure that the healthy parent is coping with all the extra chores and helping out with cleaning, washing, and care whenever possible. There is a sense of powerlessness, abandonment, and loneliness but also anger against the demented parent at how they changed and all the crazy things they do. There is also a presence of fear: fear of inheriting the disease. Additionally, there is longing for what has been (the previous relation) and missing the way you communicated with them before the disease.

### Structural analysis

The structural analysis constitutes three themes—*Being frustrated*, *Feelings of loss*, and *Being burdened*—as well as 14 subthemes, listed in Table II. The three main themes are described in the remainder of this section.

**Table II:** Structural analysis.

Subthemes	Themes
Feeling powerless	Being frustrated
Being angry and bitter	
Wanting to disconnect	
Striving for control	
Feeling resigned	
Feeling abandoned	Feelings of loss
Wanting to connect	
Feelings of sorrow	
Being compelled to take responsibility	Being burdened
Feeling worn out	
Being worried	
Wishing well	
Being the parent	
Having a bad conscience	

Subthemes, themes, and main theme that emerged from the narratives of the adult children.

#### Being frustrated

Being frustrated included feeling powerless regarding the changes taking place due to the disease in the parent and in your personal life; you can only look on as the person changes. “I brought things to stimulate her memory but she didn't remember.”

Being bitter and angry about the circumstances of your parent's disease and what it meant for the family; not being able to enjoy each other's company as you used to. “I cannot let the anger over mother's behavior affect her. She was never like this before; I swear and curse when I get home instead.”

Being frustrated was also associated with how the disease and the needs that followed of the diseased parent changed and how the involved healthcare professionals handled these issues. “I don't think about it as often but it's hard seeing dad so poorly that he needs help with everything.” There is also frustration with care organizations which don't take responsibility for diagnoses and care. “I said; I don't care who's paying just make sure he's thoroughly investigated and diagnosed, but it was never done and that's a shame.”

It also meant wanting at times to dissociate oneself from the diseased parent when things got too crazy. “She does so weird things, like putting a napkin over a cup and trying to drink through it. Why would you do that?” At the same time, they were also striving to control the situation by all means possible. “Mum has a nice accommodation now but we don't know what happens when we're not there. If we ask, nobody knows anything and they cannot tell us.” However, the adult children were on some level feeling resigned about the whole situation and the lack of control in the process. “I cannot let go despite them living in sheltered accommodation. I want to know, be in control. It has been hard.”

#### Feelings of loss

Feeling loss meant that the adult children were feeling as though their parent abandoned them and were longing to connect to their diseased parent again. “Just think if I could have mum back like she was 10 years ago or just 5. That would be so fun, but this is how it is now.” There was a sense that the parent they knew was gone and had left them behind. “It's a great loss, it's like it's not my mother. It feels like; where did you go?” One said outright, “I feel like a little kid, being abandoned (by my parent).”

Feelings of loss also meant profound feelings of sorrow: mourning the loss of the parent and missing having them in their life. “The hardest part is that you lost a parent; still exists but lost. I don't know if it would be easier if she was dead.” They missed the specific part of life that only they shared with their parent. “Mother and I could always have a giggle together, but not now.” That parent is lost and will not return—a personal loss they will have to come to terms with. “I take it day by day. It's hard sometimes, dad is stable now but it will never get any better.” The changes in the parent's personality are hard for them to witness. “Sometimes I feel like it would be for the best if she died but you don't want that either. You just want her to be released from this demeaning transformation.” The fact that the parent at times seemed aware of the changes was also hard for them. “Mum sometimes says that she will want to come home when they found out what is wrong with her.”

They longed for the accomplished person they felt their parent was before the disease—the parent they could ask for advice or lean on for support when things became hectic in their own life. “I feel sad that I can no longer discuss things with my mother or ask her things.” It was especially hard if their parent was particularly skilled in something. “Mother was so knowledgeable and skilled. She could mend clothes in a way so it became invisible.”

#### Being burdened

Being burdened meant that the adult children felt compelled to take responsibility for the diseased parent and the situation, and that no one else did. “Dad had not been diagnosed until mum got sick and ended up in hospital, and then I had to take over.”

Being burdened also meant feeling worn out due to overworking, taking care of their parents’ household as well as their own, and helping out as best they could. “When mum got sick my life was really busy with fulltime job; taking the kids to different sporting events in my spare time. It was like being on a treadmill.” The adult children were making sure that everything was as the diseased parent was used to. “Sure, it's been hard and still is. Sometimes you just don't want to think about how awful it was; just getting mum into the shower could be impossible sometimes.” The adult children were worried about their diseased parent but also about their healthy but old parent who was still living at home. “You become really tied up. You dart around like mad between work, dad, and home.”

They also worried a lot about the heredity of the disease, being afflicted by dementia themselves someday and trying to prepare mentally for that. “I wonder if I will become like mother but I don't expect to live that long.” Many feared that writing lists was a sign of dementia. “I have to write everything down myself to remember, otherwise I forget. Maybe I should be checked for dementia?”

The adult children were also burdened by having to assume a parent's role for their parent. “This has been a weird journey; she is like a little kid again. Mum, she says to me. Yes, I say, I am your mother now. It's easier that way (to get along), so you just accept it.”

Being burdened meant also wishing well for their parent, trying to make the best for them just like a parent would for their child. “I took some curtains from home and put out photos of all the grandkids with name labels on them.” At the same time, one could experience constantly having a guilty conscience for not doing more for them, despite however much they actually did. “You are content with that they are spry (physically), but it's hard. I would not wish this on my worst enemy. I go home every day feeling anxious for them. I am just that type.”

## Comprehensive understanding

To be an adult child of a person with dementia disease means being burdened with the responsibility to act on behalf of the diseased parent despite a deep sense of grief and loss, which leads to frustration with the situation.

## Discussion

The comprehensive understanding could be compared to a psychic crisis, which is defined as a situation triggered by an event that threatens the individual's physical existence, social identity, and safety, or the possibilities for basic satisfaction in existence (Cullberg, 1988). A crisis can also be described as a situation that leads to radical changes in the afflicted human's relationship to life and reality (Persson, 1995), or simply, as Caplan put it, “an upset in a steady state” (Rapoport, 1962, p. 212). Having a parent afflicted by dementia and changing before you is a dire situation that affects your sense of safety. One's existence and reality are threatened not only by the changing parent but also by the possibility of inheriting the disease. This fear of inheriting the disease is, to our knowledge, not found in other research regarding caregivers but rather is a unique trait for the adult children.

Symptoms of crises are expressed as feelings of guilt, anger, frustration, as well as abandonment; all are present in our findings. A parent afflicted by dementia and in need of help who is as well exhibiting behavior problems also causes difficulties in the relationship. Comparing the adult children's situation with research regarding stressful life events and depressive symptoms shows that among the most stressful events were problems with parents (Avison & Turner, 1988). Looking at life stress and event resolution, one of the most significant is death of family members (Turner & Avison, 1992). In this case, restoring a steady state is not possible, and dealing with the situation will lead to major changes for the individual (Loughran, 2011).

As seen in the results, some of the participants talked about their parent as if they were dead or as if they were not sure what the difference would be. That person was now lost to them, and they had to take care of themselves as well as their diseased parent. There was no way to get away or shed the responsibility for the diseased parent. Our participants expressed it as: they felt compelled to take responsibility for the well-being of their parent. Compare this to Levinas’ proposition about the Other being an unreductable part of life that forces the self to act, an asymmetrical relationship in which the Other obligates the Self to responsibility and to help (Levinas, 1969). In a way, they have no other choice than to accept the responsibility. Levinás also states that you are responsible for the Other's responsibility, that you support the Other, and are not able to shed that responsibility because you are humans (Levinás, 1990). According to Logstrup, in the human existence there is a constant demand in social norms of reciprocity; others should do unto you as you do unto them, and vice versa. This means taking care of the one who cared for you and gave you a sense of personality. This is understood here as your parent. To be a personality means that something is demanded of you and that you need to be accountable to yourself for your action or nonaction (Logstrup, 1992). Growing up, your parents gave you your sense of personality and enabled you to move out into the world as a young adult. Erikson's (1997) description of the developmental stage of Adulthood shows that a new virtue emerges in that stage, namely, Care, a widening commitment to take care of persons whom you have learned to care for. As seen by the comments in the findings, all of our participants cared deeply for their parent and were doing their best to take care of them. This also means that the adult children were, in a way, according to Erikson, transmitting ideal values to the next generation, bringing out the truest and best in loyalty and heroism, cooperation and inventiveness (Erikson, 1997), in a way caring for the next generation as well as the previous one.

In the findings, our participants expressed that they were working hard trying to take care of not just their parent but also their own family. Our participants’ experience with overworking, trying to manage two households at the same time, is also confirmed by the findings of Conde-Sala, Garre-Olmo, Turró-Garriga, Vilalta-Franch, and López-Pousa (2010). Their study of caregiver burden showed that especially daughters with other family duties showed an increased caregiver burden (*p*<0.001). Since eight of our nine respondents were female, this finding could also apply to them.

Conde-Sala et al. (2010) also showed that for a spouse to take care of a diseased spouse was considered a marital duty, but for adult children it meant a change in lifestyle. Something that all of our participants described was putting their own life on hold to take care of their parent. As Levinás states, the responsibility is mine, and as a human being I cannot refuse to take it. My nonexchangeable Me means that only I am responsible; I can assume others’ roles, but no one can assume Mine (Levinás, 1990). However, to successfully move out of crisis and be able to return to life afterward with one's resources intact or even improved, there needs to be adaptation (Persson, 1995). A crisis can be handled if the individuals adapt their current behavior, their effect, and their understanding of the crisis. This will allow them to build on their ego strengths and available resources (Loughran, 2011). According to Persson (1995), it is possible to reclaim your lost identity when you are allowed to express all your experiences and emotions. This enables you to feel understood in your despair. This could also be understood by Erikson (1997) as a meaningful crisis.

### Methodological considerations

The participants were not chosen with the greatest variety in mind, but rather a strategic sample was made from participants in an ongoing support group. However, the participants’ narratives were similar, and no one stood out in any way despite differences in diagnoses of the parents, parents’ living conditions, or ages of the interviewees. We can, however, say nothing about the situation for adult children who are not receiving support by the municipal. Their situation can be more difficult because of not receiving support, or it could be that they feel no need for support and are coping well on their own. The choice to ask the adult children in the support group was twofold: first, they were easy to get in touch with; and, second, they were used to express their experiences to people unknown to them. All the interviewees also stated at the end of the interviews that it was nice to talk about events with someone unbiased actively listening to them. When a phenomenological hermeneutic approach is used, the interviews should be in depth and the storyteller reflecting at the same time as telling their story. In a way, they are interpreting their own story while telling it to someone else (Linseth & Norberg, 2004). This is also a way to make sense of their own story, a valuable point in recovery from trauma. This study provides one interpretation of the text; other interpretations are also possible (Ricoeur, 1976). Pre-understanding constitutes an important aspect of the analysis, yet bracketing pre-understanding is never fully feasible. However, the authors discussed pre-understanding, being aware that most pre-understanding is on an unconscious level, and the impact of prejudice and assumptions focusing on being open to the phenomenon, as suggested by Lindseth and Norberg (2004). The first author has been responsible for data collection, analysis, and manuscript preparation. However, all authors have been involved in the entire research process, and the findings were thoroughly scrutinized and discussed among all authors until consensus was reached. The authors’ different experience and knowledge background contributed to trustworthiness. As mentioned in this article, the naïve reading can be described as guesswork, in this case of what it means to be an adult child of a person with dementia. This guesswork is then validated by the structural analysis. If the structural analysis does not validate the naïve reading, you start over by reading the whole text, formulate a new naïve understanding, and proceed to a new structural analysis to validate that. In our study, the first naïve reading was validated by the structural analysis as seen in this article.

## Conclusions

The findings suggest that adult children of persons with dementia are in need of support for some time to adapt to the fact that they lost a parent who is still alive. The adult children should be offered support groups as a means of crisis intervention. They should therefore have the possibility to talk about how they feel in a nonjudgmental environment with others in the same predicament in the presence of a skilled moderator who can answer any questions. They also need information about the disease and the process of diagnosis and treatment to feel like they are part of the process as well as understand the behavior and needs of their parent—to be a more skilled caretaker, as it were.

The adult children should also be included in the diagnostic stage with either specific written information for them or a special meeting with the diagnosing physician to address questions regarding prognosis, medication, and future problems. Also, the issue of the risk of inheriting the disease and what it would mean should be addressed. The care organization should also be aware of the extra work that the adult children often do to take care of both households and find a way to alleviate that burden early on to make adaptation easier.
